# Variation in the Heritability of Child Body Mass Index by Obesogenic Home Environment

**DOI:** 10.1001/jamapediatrics.2018.1508

**Published:** 2018-10-01

**Authors:** Stephanie Schrempft, Cornelia H. M. van Jaarsveld, Abigail Fisher, Moritz Herle, Andrea D. Smith, Alison Fildes, Clare H. Llewellyn

**Affiliations:** 1 Department of Behavioural Science and Health, University College London, London, United Kingdom; 2 Departments for Health Evidence and Primary and Community Care, Radboud University Medical Center, Nijmegen, the Netherlands; 3 University College London Great Ormond Street Institute of Child Health, London, United Kingdom; 4 School of Psychology, University of Leeds, Leeds, United Kingdom

## Abstract

**Importance:**

The early obesogenic home environment is consistently identified as a key influence on child weight trajectories, but little research has examined the mechanisms of that influence. Such research is essential for the effective prevention and treatment of overweight and obesity.

**Objective:**

To test behavioral susceptibility theory’s hypothesis that the heritability of body mass index (BMI) is higher among children who live in more obesogenic home environments.

**Design, Setting, and Participants:**

This study was a gene-environment interaction twin study that used cross-sectional data from 925 families (1850 twins) in the Gemini cohort (a population-based prospective cohort of twins born in England and Wales between March and December 2007). Data were analyzed from July to October 2013 and in June 2018.

**Exposures:**

Parents completed the Home Environment Interview, a comprehensive measure of the obesogenic home environment in early childhood. Three standardized composite scores were created to capture food, physical activity, and media-related influences in the home; these were summed to create an overall obesogenic risk score. The 4 composite scores were split on the mean, reflecting higher-risk and lower-risk home environments.

**Main Outcomes and Measures:**

Quantitative genetic model fitting was used to estimate heritability of age-adjusted and sex-adjusted BMI (BMI SD score, estimated using British 1990 growth reference data) for children living in lower-risk and higher-risk home environments.

**Results:**

Among 1850 twins (915 [49.5%] male and 935 [50.5%] female; mean [SD] age, 4.1 [0.4] years), the heritability of BMI SD score was significantly higher among children living in overall higher-risk home environments (86%; 95% CI, 68%-89%) compared with those living in overall lower-risk home environments (39%; 95% CI, 21%-57%). The findings were similar when examining the heritability of BMI in the separate food and physical activity environment domains.

**Conclusions and Relevance:**

These findings support the hypothesis that obesity-related genes are more strongly associated with BMI in more obesogenic home environments. Modifying the early home environment to prevent weight gain may be particularly important for children genetically at risk for obesity.

## Key Points

**Question:**

Is the heritability of body mass index higher among children who live in more obesogenic home environments?

**Findings:**

In this cohort study of 925 twin pairs, the heritability of body mass index at 4 years for those living in higher-risk obesogenic home environments was 86% and more than double that for those living in lower-risk obesogenic home environments (39%).

**Meaning:**

These results suggest that obesity-related genes are more strongly associated with body mass index in more obesogenic home environments, and that genetic predisposition to obesity could be buffered by the early home environment.

## Teaser

This cohort study investigates the association between obesogenic home environment and heritability of body mass index among children.

## Introduction

Human body mass index (BMI) (calculated as weight in kilograms divided by height in meters squared) is highly heritable, as indicated in recent reviews of twin studies.^1,2^ However, there is substantial variation in BMI heritability estimates, which range from 31% to 90%.^2^ This variation has been attributed to both population and socioenvironmental characteristics. The heritability of BMI is higher in populations with higher average BMIs,^2^ in countries with higher gross domestic product,^2^ in populations born later,^3^ and in families of lower socioeconomic status.^4,5^ These findings are in line with the hypothesis that obesity-related genes are more strongly associated with BMI in more obesogenic home environments.

Molecular genetic studies have corroborated findings from twin studies, showing that the environment modifies the association between measured genetic risk of obesity and BMI. In a large European sample of children (n = 4406), the effect of the *FTO* genotype on BMI was stronger among children with parents of low socioeconomic status.^6^ In another study, the association between a composite indicator of genetic risk of obesity and BMI was stronger for more recent birth cohorts, who by implication had had greater exposure to the obesogenic environment.^7^

Differences in economic growth and socioeconomic status are macro-level influences of the environment. The food, physical activity, and entertainment environments are proximal or micro-level influences on energy intake and physical activity; these include the home, school, and neighborhood settings.^8^ Some research has found that living in more walkable neighborhood environments suppresses genetic variance in adult BMI.^9^ However, no studies have examined whether the heritability of BMI varies by the home environment in childhood. This is an important research endeavor because the home environment is within an individual’s control and has been identified as a key influence on early weight trajectories.^10,11^ Understanding the role of the home environment from a gene-environment perspective can further inform home-based childhood obesity prevention and treatment efforts, which have been ineffective.^12^

The obesogenic home environment incorporates food, physical activity, and media-related influences, such as the availability of healthy and unhealthy foods, opportunities for physical activity, and parental rules around media use.^13,14^ Any single aspect of the home environment probably has limited influence on weight-related outcomes; therefore, composite measures should capture overall obesogenic risk most effectively. Recent findings have shown that preschool children who lived in higher-risk home environments, as measured by the Home Environment Interview (HEI) (the sum of 21 food-related, 6 physical activity-related, and 5 media-related factors), had poorer diets, engaged in less physical activity, and watched more television than did children who lived in lower-risk home environments.^15^

This study expands previous research by examining whether the heritability of child BMI varies by the early obesogenic home environment. It is hypothesized that the heritability of BMI will be higher among children living in higher-risk home environments compared with those living in lower-risk home environments.

## Methods

### Sample

Gemini cohort data (a nationally representative twin study of early growth^16^) were used in this study. In total, 2402 of 6754 families (36% of those with live twin births in England and Wales during March-December 2007) gave written consent to participate and completed a baseline questionnaire when their children were a mean (SD) of 8.2 (2.2) months of age (range, 4–20 months). The HEI was completed by 1113 of 2402 families (46% of the total sample) when the children were a mean (SD) of 4.2 (0.4) years of age (range, 3–5 years). This study sample comprised 925 twin pairs (1850 twins) with data on all study variables. Data were analyzed from July to October 2013 and in June 2018. Ethical approval was granted by the University College London Committee for the Ethics of non–National Health Service Human Research. Data were deidentified.

### Measures

#### Zygosity

Opposite-sex twins were classified as dizygotic (DZ). Parents of same-sex twins were asked to complete a previously validated 20-item zygosity questionnaire,^17^ which assesses the twins’ physical likeness, blood type, how easily friends and family members can tell the twins apart, and parents and health professionals’ opinions about the twins’ zygosity. The questionnaire showed 100% agreement with DNA samples of 81 randomly selected Gemini twin pairs (43 monozygotic [MZ] twins and 38 DZ twins) at 29 months of age.^18^

#### Body Mass Index

Electronic weighing scales and height charts were sent to all families when the twins were 2 years of age to collect parent-reported measurements every 3 months. Parents also provided their twins’ heights and weights at the time of the HEI. The BMI SD scores, adjusted for age and sex, were calculated using British 1990 growth reference data^19^ and the LMS growth macro for Excel (Microsoft Corporation).

#### Home Environment

Primary caregivers (1102 of 1113 caregivers [99%] were mothers) completed the HEI by telephone when their twins were 4 years of age. The HEI is a comprehensive home environment measure assessing food, physical activity, and media-related influences.^15^

As described elsewhere,^15^ the level of obesogenic risk was determined by creating composite scores, guided by feedback from an international panel of 30 experts in pediatric obesity. A total of 32 constructs were included in the composites (eTable 1 in the Supplement). Constructs associated with lower risk of excessive weight gain were reverse-scored so that higher total scores would reflect higher obesogenic risk. Each variable was standardized using *z* scores and summed to create composite scores for the home food environment (21 variables), the home activity environment (6 variables), and the home media environment (5 variables). There were few cases with missing data on home environment variables; these were recoded to 0 (the mean value for each standardized variable). The 3 composites were summed to create an overall home environment composite, dividing by the number of variables per composite so that each domain contributed equally to the overall score (food composite/21 + activity composite/6 + media composite/5).

Test-retest reliability of the home environment composites from 7 to 19 days (mean [SD], 9.6 [3.4] days) was acceptable to high. The intraclass correlation coefficients were 0.71 (95% CI, 0.52–0.83) for food, 0.83 (95% CI, 0.72–0.91) for activity, 0.92 (95% CI, 0.85–0.95) for media, and 0.92 (95% CI, 0.86–0.96) overall.

An overview of the measurement points is given in eTable 2 in the Supplement.

### Statistical Analyses

#### Heritability Analyses

Genetic and environmental contributions to variation in a trait can be estimated by comparing similarity between MZ twins (who share 100% of their genes) with that between DZ twins (who share approximately 50% of their genes). Comparing MZ and DZ correlations enables variation in a trait to be decomposed into 3 latent factors (the ACE model): additive genetic effects (ie, heritability) (A); shared environmental influence (shared experiences that make twins within a pair similar) (C); and nonshared environmental influence (experiences unique to an individual that make twins within a pair different) (E), which also includes random measurement error.^20^

Two methods were used to estimate the heritability of BMI at 4 years of age: twin correlations and maximum likelihood structural equation modeling (MLSEM).^21^ For each method, 4-year BMI SD score was residualized for age at BMI measurement and sex effects using linear regression.^22^ The analyses were repeated using BMI SD scores additionally residualized for gestational age, which is also exactly correlated within twin pairs.

Heritability estimates for 4-year BMI SD scores were calculated for the total sample and for home environment groups dichotomized on the mean (0): lower (≤0) and higher (>0) overall risk, food, activity, and media home environments.

#### Twin Correlations

Intraclass correlations were calculated for each zygosity (MZ and DZ) and for each zygosity by each home environment group (eg, MZs living in a home environment with higher overall risk) in R^23^ using the structural equation modeling software OpenMx, version 2.2.6.^24^

#### Model Fitting

Univariate twin models were created in R^23^ using the structural equation modeling software OpenMx, version 2.2.6^24^ to produce reliable parameter estimates for the whole sample with 95% CIs and goodness-of-fit statistics. A heterogeneity model was used to test for differences in the magnitude of A, C, and E between the lower-risk and higher-risk home environment groups (eFigure in the Supplement). A, C, and E were estimated using the covariance between twins. Because MZs share 100% of their genes and DZs share approximately 50% of their genes, the genetic correlations within MZ and DZ pairs were fixed at 1.0 and 0.5, respectively. Because it is assumed that shared environmental influences are equal for MZ and DZ twins, the shared environmental correlation was fixed at 1.0 for both zygosities.

A common effects model was fitted to compare parameter estimates in lower-risk and higher-risk home environment groups. This model allows the magnitude of variance explained by A, C, and E to differ between groups. The fit of more constrained nested models was then compared with the original model using likelihood ratio tests. A significant difference between the negative log-likelihood of the nested model and that of the original model indicates a deterioration in model fit.^25,26^ The 2 nested models in this study were the scalar model, which allows variance differences but not quantitative differences between groups, and the null model, which constrains all parameters to be the same across the 2 groups. If the scalar or null models show a better fit than the common effects model, there are no quantitative differences in parameter estimates between groups.^25,26^ Statistical significance was set at .05, and *P* values were 1-sided.

## Results

### Sample Characteristics

Of the total HEI sample (1113 families; 2226 twins), 12 twin-pairs had unknown zygosity, and 174 first-born twins and 177 second-born twins had missing data for 4-year BMI. This left a sample of 925 twin pairs (1850 twins; 915 [49.5%] male and 935 [50.5%] female; mean [SD] age, 4.1 [0.4] years). There were no significant differences between the study sample and the total HEI sample with respect to the study variables (eTable 3 in the Supplement).

Three hundred fourteen of 925 twin pairs (34%) were MZ. There were slightly more twin pairs living in lower-risk home environments than higher-risk homes (508 [56%] vs 417 [46%]). Mean (SD) 4-year BMI SD score was below that of the reference population (first-born twins: −0.01 [1.03]; second-born twins: −0.10 [1.03]). The ranges for the home environment composites (standardized scores) showed that there was substantial variation (overall, −2.44 to 4.02; food, −19.24 to 25.24; activity, −4.93 to 16.15; media, −7.00 to 18.12). Sample characteristics by higher-risk and lower-risk home environments (overall) are shown in Table 1. Families living in higher-risk home environments had significantly higher risk scores for each of the food (*t*_838_ = −19.35; *P* < .001), physical activity (*t*_683.44_ = −18.85; *P* < .001), and media (*t*_628.05_ = −18.73; *P* < .001) environment composites compared with those living in lower-risk home environments. The proportion of university-educated mothers (χ^2^_2_ = 31.57) and families with professional occupations (χ^2^_2_ = 26.70) was significantly smaller among those living in higher-risk home environments (*P* < .001).

**Table 1.** Characteristics of the Study Sample by Overall Home Environment Risk

Characteristics	Overall Higher-Risk Home Environment (n = 417)	Overall Lower-Risk Home Environment (n = 508)	*P* Value Difference^a^
Age at HEI, mean (SD), y	4.13 (0.44)	4.16 (0.37)	.19
Sex of twin pair, No. (%)			
Male	147 (35.3)	167 (32.9)	.74
Female	144 (34.5)	180 (35.4)	
Opposite sex	126 (30.2)	161 (31.7)	
Zygosity, No. (%)			
Monozygotic	151 (36.2)	163 (32.1)	.19
Dizygotic	266 (63.8)	345 (67.9)	
Maternal educational level, No. (%)^b^			
Low	80 (19.2)	56 (11.0)	<.001
Medium	170 (40.8)	157 (30.9)	
High	167 (40.0)	295 (58.1)	
NSSEC, No. (%)^c^			
Low	75 (18.0)	46 (9.1)	<.001
Medium	76 (18.3)	62 (12.2)	
High	265 (63.7)	399 (78.7)	
Composite score, mean (range)	0.81 (−0.03 to 4.02)	−0.70 (−2.44 to −0.03)	<.001
Food score, mean (range)	3.84 (−11.35 to 25.24)	−3.09 (−19.24 to 9.46)	<.001
Activity score, mean (range)	1.85 (−4.93 to 16.15)	−1.49 (−4.93 to 5.79)	<.001
Media score, mean (range)	1.86 (−6.45 to 18.12)	−1.81 (−7.00 to 4.37)	<.001
4-y BMI SD score, mean (SD)	−0.06 (1.05)	−0.02 (0.99)	.57

Abbreviations: BMI, body mass index (calculated as weight in kilograms divided by height in meters squared); HEI, Home Environment Interview; NSSEC, National Statistics Socio-economic Classification.

^a^ Characteristics of those living in higher-risk vs lower-risk home environments were compared using χ^2^ for categorical variables and *t* tests for continuously distributed variables. One twin was selected at random to avoid clustering effects.

^b^ Educational level categorized as low (no qualifications or basic high school education), medium (vocational or advanced high school education), and high (university-level education).

^c^ NSSEC level categorized as low (lower supervisory and technical occupations, routine or semiroutine occupations, never worked, and long-term unemployed), medium (intermediate occupations, small employers, and own-account workers), and high (higher and lower managerial and professional occupations).

### Twin Correlations

The intraclass correlation coefficients for 4-year BMI SD score (adjusted for age and sex) by zygosity and home environment groups are shown in Table 2. Correlations were higher between MZ than DZ twins (ranges, 0.78-0.87 vs 0.37-0.54), indicating additive genetic variation in BMI. The size of the difference between MZ and DZ twins varied by the level of home environment risk, with greater differences in higher-risk than lower-risk home environments (overall, 0.46 vs 0.27; food, 0.43 vs 0.28; activity, 0.46 vs 0.27), although the difference was smaller between higher-risk and lower-risk media environments (0.39 vs 0.32). The results were the same when additionally adjusting 4-year BMI SD score for gestational age.

**Table 2.** Intraclass Correlations of BMI SD Score at 4 Years by Zygosity and Home Environment Risk

Home Environment Risk Group	No. (%) of Twin Pairs		Intraclass Correlation Coefficient (95% CI)	
	MZ (n = 314)	DZ (n = 611)	MZ	DZ
Overall home environment				
Lower risk	166 (52.9)	351 (57.4)	0.78 (0.71-0.83)	0.51 (0.43-0.58)
Higher risk	148 (47.1)	260 (42.6)	0.87 (0.83-0.91)	0.41 (0.31-0.51)
Home food environment				
Lower risk	146 (46.5)	333 (54.5)	0.80 (0.73-0.85)	0.52 (0.44-0.59)
Higher risk	168 (53.5)	278 (45.5)	0.84 (0.79-0.88)	0.41 (0.31-0.50)
Home activity environment				
Lower risk	179 (57.0)	350 (57.3)	0.81 (0.76-0.86)	0.54 (0.46-0.61)
Higher risk	135 (53.0)	261 (42.7)	0.83 (0.77-0.88)	0.37 (0.26-0.47)
Home media environment				
Lower risk	174 (55.4)	375 (61.4)	0.80 (0.74-0.85)	0.48 (0.40-0.55)
Higher risk	140 (44.6)	236 (38.6)	0.84 (0.78-0.88)	0.45 (0.35-0.55)

Abbreviations: BMI, body mass index (calculated as weight in kilograms divided by height in meters squared); DZ, dizygotic; MZ, monozygotic.

### Maximum Likelihood Structural Equation Modeling

For the total sample, variance in BMI was largely attributable to additive genetic factors (62%; 95% CI, 49%-75%), moderately attributable to shared environmental factors (18%; 95% CI, 5%-29%), and moderately attributable to nonshared environmental factors (20%; 95% CI, 17%-24%). Parameter estimates for higher-risk and lower-risk home environments are summarized in Table 3. For the overall home environment, the common effects model gave the best fit to the data, indicating that the heritability of BMI SD score was significantly and substantially higher (86% vs 39%) in higher-risk home environments. There was also a difference in the proportion of variance in 4-year BMI SD score attributable to shared environmental factors across the 2 groups; 34% for lower-risk home environments and 0% for higher-risk home environments. For the home food and media environments, the common effects model also provided the best fit to the data. For the home physical activity environment, there were observable differences in the parameter estimates for the higher-risk and lower-risk groups. However, the scalar model was not a significantly worse fit to the data than the common effects model, and a null model did not fit the data well. This indicated that there were significant differences in variances across the higher-risk and lower-risk groups. These results were replicated when additionally adjusting 4-year BMI SD score for gestational age.

**Table 3.** Parameter Estimates and Goodness-of-Fit Statistics for Home Environment Interaction Models That Examined the Heritability of BMI SD Score at 4 Years of Age^a^

Home Environment, Model^b^	Estimate			Change in AIC	*P* Value^d^
	Additive Genetic	Environment			
		Shared	Nonshared^c^		
Overall					
Common effects					
Lower risk	0.39 (0.21-0.57)	0.34 (0.18-0.49)	0.27 (0.21-0.33)	NA	NA
Higher risk	0.86 (0.68-0.89)	0.00 (0.00-0.17)	0.14 (0.11-0.18)	NA	NA
Scalar	0.62 (0.49-0.75)	0.18 (0.05-0.29)	0.20 (0.17-0.24)	15.183	<.001
Null	0.62 (0.49-0.75)	0.18 (0.05-0.29)	0.20 (0.17-0.24)	−1.524	.49
Food					
Common effects					
Lower risk	0.40 (0.23-0.58)	0.35 (0.18-0.49)	0.25 (0.20-0.31)	NA	NA
Higher risk	0.83 (0.65-0.87)	0.00 (0.00-0.18)	0.17 (0.13-0.21)	NA	NA
Scalar	0.62 (0.49-0.76)	0.18 (0.05-0.29)	0.20 (0.17-0.24)	6.693	.005
Null	0.62 (0.49-0.75)	0.18 (0.05-0.29)	0.20 (0.17-0.24)	−1.446	.46
Activity					
Common effects					
Lower risk	0.49 (0.33-0.65)	0.31 (0.15-0.44)	0.21 (0.17-0.26)	NA	NA
Higher risk	0.80 (0.60-0.84)	0.00 (0.00-0.00)	0.20 (0.16-0.26)	NA	NA
Scalar	0.62 (0.49-0.75)	0.18 (0.05-0.29)	0.20 (0.17-0.24)	0.288	.10
Null	0.62 (0.49-0.75)	0.18 (0.05-0.29)	0.20 (0.17-0.24)	−1.987	.91
Media					
Common effects					
Lower risk	0.60 (0.42-0.78)	0.18 (0.01-0.33)	0.23 (0.18-0.29)	NA	NA
Higher risk	0.65 (0.46-0.84)	0.17 (0.00-0.34)	0.18 (0.14-0.23)	NA	NA
Scalar	0.62 (0.49-0.76)	0.18 (0.05-0.29)	0.20 (0.17-0.24)	9.123	.002
Null	0.62 (0.49-0.75)	0.18 (0.05-0.29)	0.20 (0.17-0.24)	−1.002	.32

Abbreviations: AIC, Akaike information criterion; BMI, body mass index (calculated as weight in kilograms divided by height in meters squared); NA, not applicable.

^a^ The BMI SD scores modeled were residuals adjusted for age at BMI measurement and sex. Presented models include all children with valid data for age, sex, Home Environment Interview score, and 4-year BMI SD score. An additional 7 cases in which just 1 twin within the pair had available BMI data were included in the maximum-likelihood structural equation modeling, performed with OpenMx software, version 2.2.6.

^b^ Statistical analyses: standard ACE model-fitting analyses for continuous data were used to model BMI SD score at 4 years of age.

^c^ Includes measurement error.

^d^ *P* values were based on the likelihood ratio test and AIC. A better-fitting submodel showed a change in χ^2^ that did not represent a significant worsening of fit designated by the *P* value.

## Discussion

This is the first study, to our knowledge, to test behavioral susceptibility theory’s hypothesis that the heritability of BMI will be higher among children who live in more obesogenic home environments. As hypothesized, heritability of BMI was higher among children living in overall higher-risk home environments compared with those living in lower-risk home environments. The modeling indicated that none of the variance in BMI was attributable to shared environmental factors in the higher-risk group. In contrast, a similar proportion of the variance in BMI was attributable to shared environmental factors and additive genetic factors in the lower-risk group. The findings were similar when examining the heritability of BMI in the separate food and physical activity environment domains.

For the total sample, 62% (95% CI, 49%-75%) of the variance in 4-year BMI SD score was attributable to additive genetic factors, 18% (95% CI, 5%-29%) to shared environmental factors, and 20% (95% CI, 17%-24%) to nonshared environmental factors. These estimates largely concur with previous studies of 4-year-old children.^27^ The heritability of BMI increases throughout childhood,^27,28,29^ perhaps as individuals seek out environments in line with their genotype and allow it to be expressed freely (active gene-environment correlation)^30^ or because gene expression changes developmentally.^31^

This study builds on earlier findings that the heritability of BMI is higher in populations with higher average BMIs, with higher levels of gross domestic product, and with lower socioeconomic status.^2^ Examining the role of proximal environmental exposures is important because these factors are within an individual’s control, and it is easier to hypothesize about their potential association with neurobiological pathways that mediate the development of overweight and obesity.^32^

According to behavioral susceptibility theory,^33,34,35^ an individual’s appetitive traits confer differential susceptibility to the obesogenic environment. Individuals who have high food responsiveness and low sensitivity to satiety are more likely to overeat when there is increased opportunity to do so.^33,34,35^ Appetitive traits play a causal role in the development of weight,^36,37^ they are highly heritable,^38,39^ and they explain part of the association between obesity-related genes and weight.^40^ Many weight-related genes are highly expressed in the hypothalamus, a key regulator of appetite and food intake.^41^ Evidence also indicates that food intake is influenced by brain regions related to reward sensitivity and incentive motivation.^42,43^ It is feasible that a home environment with multiple food cues triggers appetitive and reward-related pathways, which prompt increased food intake and, subsequently, weight gain. In line with this idea, children with the *FTO* polymorphism associated with obesity risk had stronger responses to food commercials in the nucleus accumbens, a reward-related brain region,^44^ and they were more likely to consume excess calories.^45^ Physical activity suppresses the effect of obesity-related genes on BMI, perhaps also via appetitive and reward-related pathways.^46,47^ Future research should directly examine whether the home environment moderates genetic influence on BMI using a genetic risk score, because BMI is a highly polygenic trait.^48,49^

Although there were large observable differences in parameter estimates when comparing higher-risk and lower-risk home physical activity environments (80% vs 49% for variance attributable to additive genetic factors), the model-fitting indicated that the 2 groups could be combined, with no significant worsening of fit. Significant differences may emerge in larger, higher powered samples and in more extreme home physical activity environments, because there was a skew toward lower risk in this sample.^50,51^ Of note, although the common effects model provided the best fit for the home media environment data, the differences in parameter estimates when comparing higher-risk and lower-risk groups were substantially smaller than those observed for the overall environment and food domain (65% vs 60% for variance attributable to additive genetic factors). There was no difference in the proportion of variance in BMI attributable to shared environmental factors across the higher-risk and lower-risk groups (17% vs 18%). It is therefore questionable that the differences observed for the home media environment are meaningful. It is possible that gene-environment effects of the home media environment are stronger in more extreme environments^50,51^ and later in development, when media influences are more prominent.^52^ Research should further examine gene-environment effects of the separate food, physical activity, and media domains in larger and more diverse samples to clarify their relative contributions.

### Limitations

Although the findings suggest gene-environment interaction, they may be partly explained by gene-environment correlation.^30,53^ For example, a child may be born into a home environment that is correlated with their genotype (passive gene-environment correlation), and some aspects of the home environment, such as parental feeding practices, may be responsive to the child’s genotype (reactive gene-environment correlation). Models have been developed to take into account gene-environment correlation effects,^54^ but larger sample sizes are needed than that available in this study.

There are also some limitations of the twin method, which may lead to overestimation of heritability estimates. The assumption of equal shared environments among DZ and MZ twins has been challenged by individuals who believe that MZ twins experience environments that are more similar than those experienced by DZ twins.^55,56^ There is also evidence that the prenatal environment may make MZ twins less similar than the twin method assumes.^57^ However, studying twins reared apart overcomes the equal environments assumption, and principal findings match those reported in twin modeling studies.^58^ Twins are less representative of the general population than singletons in several ways, including their growth^59^; however, there is no evidence that growth patterns differ between MZ and DZ twins, which would compromise findings from twin studies.

Although it is not clear whether or how gene-environment interaction would vary by race/ethnicity, some research suggests that heritability of BMI is higher among white adolescents than East Asian adolescents.^60^ It would therefore be informative to replicate our findings in an ethnically diverse sample. Finally, as in other cohort studies, heritability estimates were derived from parent reports of height and weight. However, research supports the validity of parent-reported BMI, especially when the measures are taken at home, as in this study.^61^

## Conclusions

This is the first study, to our knowledge, to examine whether the heritability of child BMI varies by the extent to which the early home environment is obesogenic. Heritability of BMI was higher in higher-risk home environments, which supports the theory that obesity-related genes are more strongly associated with BMI in more obesogenic environments and suggests pathways through which macro-level factors, such as socioeconomic status, are associated with obesity. These findings provide further insight into the mechanisms underlying overweight and obesity and how they may be prevented.

## Notes

Supplement. **eTable 1.** Constructs included in the home environment composite scores **eTable 2.** Overview of the study measures and assessment points in Gemini **eTable 3.** Comparison of the study sample and the total HEI sample on the study variables **eFigure 1.** Heterogeneity model for higher-risk and lower-risk obesogenic home environments
