# Supplementary material for: Variation in the Heritability of Child Body Mass Index by Obesogenic Home Environment
Source: JAMA Pediatr. 2018 Oct 1;172(12):1153–60. doi: 10.1001/jamapediatrics.2018.1508 (PMC6396810; doi:10.1001/jamapediatrics.2018.1508)
Supplement: Supplement. — eTable 1. Constructs included in the home environment composite scores eTable 2. Overview of the study measures and assessment points in Gemini eTable 3. Comparison of the study sample and the total HEI sample on the study variables eFigure 1. Heterogeneity model for higher-risk and lower-risk obesogenic home environments [file jamapediatr-172-1153-s001.pdf]

Supplementary Online Content

Schrempft S, van Jaarsveld CHM, Fisher A, et al. Variation in the heritability of child body mass index by obesogenic home environment. *JAMA Pediatr*. Published online October 1, 2018. doi:10.1001/jamapediatrics.2018.1508

eTable 1. Constructs included in the home environment composite scores

eTable 2. Overview of the study measures and assessment points in Gemini

eTable 3. Comparison of the study sample and the total HEI sample on the study variables

eFigure 1. Heterogeneity model for higher-risk and lower-risk obesogenic home environments

This supplementary material has been provided by the authors to give readers additional information about their work.

© 2018 Schrempft S et al. *JAMA Pediatrics.*

eTable 1. Constructs included in the home environment composite scores

Food-related constructs
Availability
Number of fruit types[1]
Number of vegetable types[1]
Number of energy-dense snack types
Presence of sugar-sweetened drinks
Accessibility (visibility)
Fruit on display[1]
Vegetables ready-to-eat[1]
Energy-dense snacks on display
Sugar-sweetened drinks on display
Accessibility (child can help him/herself)
Fruit[1]
Vegetables[1]
Energy-dense snacks
Sugar-sweetened drinks
Parental feeding practices
Emotional feeding
Instrumental feeding
Encouragement[1]
Modelling[1]
Monitoring[1]
Covert restriction[1]
Restriction[1]
Family meal frequency
Frequency child eats while watching TV
**Physical activity-related constructs**
Garden/outdoor space[1]
Garden play equipment[1]
Allowed to play indoors[1]
Allowed to play outdoors[1]
Parental modelling of physical activity[1]
Parental support of physical activity[1]
**Media-related constructs**
Number of media equipment
TV in the child's bedroom
Household rules around media use[1]
Maternal TV viewing
Partner TV viewing

[1] Variable was identified as being associated with decreased risk for weight gain.

© 2018 Schrempft S et al. *JAMA Pediatrics.*

eTable 2. Overview of the study measures and assessment points in Gemini

	Child age in months (measurement point)							
	**8 (T0)**	**15 (T1)**	**20 (T2)**	**24 (T3)**	**30 (T4)**	**36 (T5)**	**48 (T6)**	**60 (T7)**
Home environment							X	
Sex of twin pair	X							
Zygosity	X				X			
DNA collection using cheek swab					X			
Gestational age	X							
Anthropometrics	X	X		X		X	X	X

© 2018 Schrempft S et al. *JAMA Pediatrics.*

eTable 3. Comparison of the study sample and the total HEI sample on the study variables

	Study Sample, % of Families (No.) (n = 925)	Total HEI Sample, % of Families (No.) (n = 1113)	P difference[a]
**Overall home environment**			0.738
Lower risk	54.9 (508)	54.2 (603)	
Higher risk	45.1 (417)	45.8 (510)	
**Home food environment**			0.915
Lower risk	51.8 (479)	52.0 (579)	
Higher risk	48.2 (446)	48.0 (534)	
**Home activity environment**			0.984
Lower risk	57.2 (529)	57.2 (637)	
Higher risk	42.8 (396)	42.8 (476)	
**Home media environment**			0.426
Lower risk	57.7 (534)	56.0 (623)	
Higher risk	42.3 (391)	44.0 (490)	
**Sex of twin pair**			0.986
Male	33.9 (314)	33.7 (375)	
Female	35.0 (324)	35.0 (389)	
Opposite sex	31.0 (287)	31.4 (349)	
**Zygosity**			0.957
Monozygotic	33.9 (314)	33.7 (375)	
Dizygotic	66.1 (611)	65.2 (726)	
Uknown	-	1.1 (12)	
**Gestational age (weeks), mean (SD)**	36.3 (2.47)	36.2 (2.54)[b]	0.432
**Age at BMI measurement (years), mean (SD)**	3.8 (0.35)	3.8 (0.35)[c]	1.000
**4-year BMI SDS, mean (SD)**	-0.04 (1.02)	-0.04 (1.02)[d]	0.995

HEI = Home Environment Interview; BMI = body mass index; SD = standard deviation.

[a]The characteristics of those living in higher- versus lower-risk home environments were compared using X^2 for categorical variables and t-tests for continuously distributed variables. One twin was selected at random to avoid clustering effects.

[b] N = 1106 (7 missing cases).

[c] N = 937 (176 missing cases).

[d] N = 936 (177 missing cases).

© 2018 Schrempft S et al. *JAMA Pediatrics.*

eFigure 1. Heterogeneity model for higher-risk and lower-risk obesogenic home environments

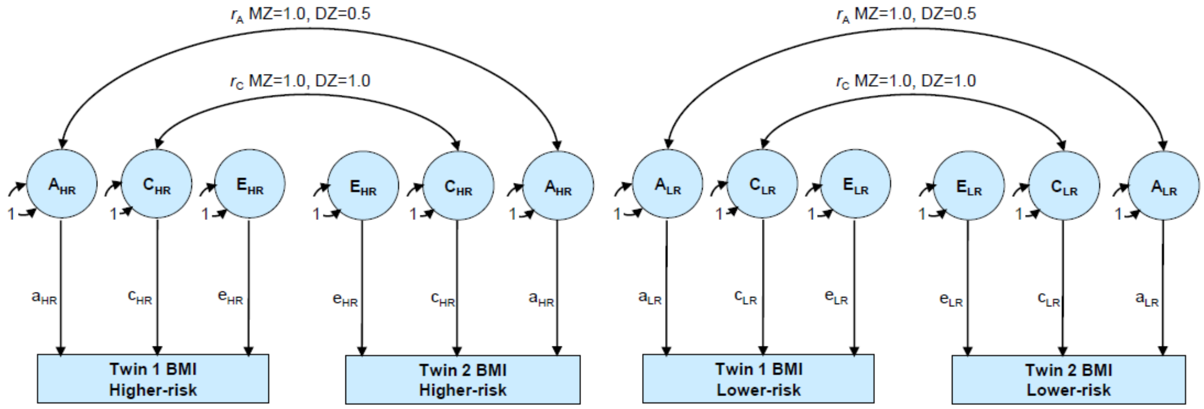

The circles indicate latent influences on measured BMI for each twin, which include additive genetic effects (A), shared environmental effects (C), and unique environmental effects (E) for twin pairs in higher-risk and lower-risk home environments separately (subscript HR or LR, respectively), which permits the evaluation of differences in the effect sizes of the parameters by home environment. The straight single-headed arrows show the causal paths (path coefficients a, c, and e) which indicate the relative influence of the latent variables on measured BMI for twin pairs in higher-risk and lower-risk environments separately (subscript HR or LR, respectively); the square of each of the path coefficients provides the variance explained for each parameter. r_A is the genetic correlation for DZ and MZ twin pairs (MZ=1.0, DZ=0.5) and r_C is the shared environmental correlation for DZ and MZ twin pairs (MZ and DZ=1.0).

© 2018 Schrempft S et al. *JAMA Pediatrics.*
